# Prevention of Disease

**Published:** 1919-06

**Authors:** 


### PREVENTION OF DISEASE

BY A PHYSICIAN

The Ministry of Health must teach the public by constant propaganda how to keep well. Prevention must begin with the expecting mother. She must be under medical and general supervision. The country must see to it that every baby possible is breast-fed. The children must be watched continuously, and their parents instructed. There is one disease above all others on which the public are urged to concentrate their attention, and that is decay of the teeth. It affects almost every member of the community. It begins in childhood. It lowers the health of the child, and consequently it predisposes it to tuberculosis and disease generally. It is impossible to exaggerate the suffering and economic loss caused to the community by this apparently trivial, but really terrible, disease. It is estimated that at least twenty per cent. of all chronic disease in this country is due, directly or indirectly, to the state of the teeth. The cost to the country through the consequent inefficiency of its citizens amounts to an enormous sum every year. The disease affects especially the British Empire and the U. S. A. For instance, *The Times* correspondent at Wellington, N. Z., sends the following message, July 16, 1918: "The President of the Dental Association was the spokesman of a deputation which urged the Government to establish dental bursaries. He said that the examination of thousands of school children in N. Z. showed the percentage of those suffering from dental caries to be ninety-five. *If cows were similarly affected they would not be tolerated for one month.*" (Italics mine.) The sting lies, as usual, in the tail. If the cows could neither crop the grass nor chew the cud it would spell ruin; and no one would rest until *the cause* was discovered. It must be obvious to any sane individual that the first essential to health is a clean, wholesome mouth, with two rows of sound teeth. Yet this necessary condition is rarely seen in our midst. The tooth, the hardest part of the body, rots while the child is in the nursery and the schoolroom. What is the object of spending huge sums on sanatoria and other schemes while this disease is allowed to exist from childhood?

Just consider how many diseases affect some part of the alimentary canal, and it will be at once obvious how necessary it is to begin with the care of the mouth. Diphtheria, tonsilitis, the sore throat of measles and scarlet fever, dyspepsia, ulcer of the stomach, appendicitis, forms of rheumatism and cancer, are examples. One of the best-known surgeons in the world actually removes *several feet* of the large intestine because the food taken into the mouth does not pass satisfactorily through the canal. Many surgeons are performing grave operations because of the ulcers existing in the stomach or the portion of the tube next below it. The public should remember that the care of the mouth is *presumed* to be in the hands of the dentist. As a matter of fact, his advice is only sought after the disease is already present. The dentist is still regarded by many of the public and of the medical profession as holding an inferior position to the latter. In reality, the dentist is a medical specialist, a dental surgeon, and of far greater importance to the community than the throat specialist. Dr. Sim Wallace, the dental surgeon, has pointed out for many years that dental disease is caused by faulty food and feeding. He claims that he is bringing up children with sound teeth today; that it is easy to do so, and that the feeding necessary is *actually cheaper.* The medical profession, for the most part, does not appreciate the fact; and, therefore, this appeal is made to the thinking public. The writer is convinced that Dr. Sim Wallace is right, and that his work will confer upon the community a greater boon than the work of any other living man. The people themselves can not possibly decide the matter; and therefore they are urged to recognize the overwhelming importance of the subject and to demand a Government inquiry into the cause of this widespread disease. Dr. Sim Wallace claims that the chief cause of the disease is the adhesive and easily fermentable nature of the modern diet; also meals between meals, and especially the bread and milk at bed-time. The last-named leaves a poultice over the crevices of, and between, the teeth during the night; nascent lactic acid arises on the tooth through the fermentation of the carbohydrate by means of bacilli; and the special affinity of this acid for the carbonates of the enamel starts the disease.

Take the principal foods of children in this country—*i. e.,* cow's milk, manufactured sugar and sweets, soft white bread, and finely milled flour in biscuits. Cow's milk belongs to the calf; and milk is the natural food for the toothless young only. In small quantities and well diluted, no doubt, cow's milk is useful in the difficult stage between the weaning of the child and the completion of its first teeth. It, or some other animal's milk, is necessary where the mother can not nurse her baby. It is not the food for healthy children with teeth. Manufactured sugar, in the enormous quantities consumed in this country in 1913, was a modern article of diet. Never grown here, the consumption of refined sugar per head of population had gone up from 9.91 lb. in 1883 to 52.23 lb. in 1900. This takes no account of the syrup in tinned fruits (Sim Wallace). There is all the difference in the world between the native sucking and chewing the *fibrous* cane and the concentrated article in the sugar basin. Prior to this invasion of sugar and sweets our children made their own sugar out of the starch in such foods as bread and potatoes. A small dose of strychnine well diluted is an excellent tonic for a convalescent; a sufficiently large dose and the undertaker is sent for. The soft white bread and fine-flour biscuits are a curse to children. It is most important to note that children for some time now have got on quite well without any white bread or fine-flour biscuits, very little sugar or sweets, and much less milk. If the diet taken by children has the effect of destroying the hardest part of the body, like rust eats into iron, it is surely permissible to suggest that the same diet may be the cause of much of the disease of the rest of the alimentary canal, and, indeed, of the body generally. Dental disease is rampant in the U. S. A.; the very fact of the number and eminence of their dental surgeons is sufficient proof. But in spite of their patching up the teeth as soon as they are diseased, appendicitis, dyspepsia, and other diseases of the alimentary canal are notoriously rife amongst them. They are simply treating the disease; they are not practicing *prevention.*

Let us, *at a cost of less than nothing to the exchequer,* start a race of children fed on rational grounds, with sound teeth and healthy mouths, and then see what happens to them. It is the writer's firm belief that appendicitis and gastric ulcer will become rarities. The writer of this article, prior to August, 1914, was in charge of a hospital for poor country Arabs. As a whole, the country Arab, as opposed to the town Arab, had very good teeth. His diet was composed of a course, hard, whole-meal bread; his staple food, eggs, fish, meat when he could afford it, butter, and a large amount of fresh fruit, and dried in the shape of dates. He took no sugar beyond what was in the fruits. In several years not one case of appendicitis was seen; not one case of gastric ulcer, no tonsils or adenoids; cancer was almost unknown, not one case of cancer affectany part of the alimentary canal. The writer then returned to England, where appendicitis is a scourge, dyspepsia and gastric ulcer rife, tonsils and adenoids the same, where cancer is all too prevalent, in order to look after the *picked* young men of the race. In an examination of the mouths of thousands of soldiers *one* perfectly sound set of teeth was discovered. On the other hand, tooth-plates were quite common in lads of nineteen and upwards; decayed teeth and missing teeth the absolute rule. Thousands were rejected from the army because of their teeth, or because of disease due to them. The condition of the young women is known to be as bad, if not worse. In conclusion: (1) Reform the diet at once. Give the children three meals a day and nothing between meals. Give them coarse, whole-meal bread. No sweets at all. Finish each meal with something cleansing, such as dry toast and butter, crust of bread and butter or margarine, celery or fruit. Give children a piece of apple after the last meal to cleanse their mouth for the night. Spoil them with fruit and toys, but never with sweets. Grow more apples in the country. (2) Develop the carriage, cold-storage and distribution by motor to every cottage in the country of our wonderful fish supply, eating and paying for far less inferior imported butcher's meat. Meat was almost unknown to the poor of England prior to the recent cold-storage invasion. Each cottager killed his pig and tasted no beef or mutton. Far too much meat was being consumed here in 1913 by those who could afford it. (3) Destroy at once the rats which consume our food and spread disease, including plague, amongst us. (4) Muzzle all dogs throughout the country for twelve months, and stamp out the rabies which has broken out in Devon and Cornwall before it is too late.—*Statist* and *British Dental Record.*

